# Syndromic case definitions for lower respiratory tract infection (LRTI) are less sensitive in older age: an analysis of symptoms among hospitalised adults

**DOI:** 10.1186/s12879-024-09425-7

**Published:** 2024-06-07

**Authors:** Rachel Kwiatkowska, Anastasia Chatzilena, Jade King, Madeleine Clout, Serena McGuinness, Nick Maskell, Jennifer Oliver, Robert Challen, Matthew Hickman, Adam Finn, Catherine Hyams, Leon Danon, the AvonCAP Research Group, Madeleine Clout, Anna Morley, Amelia Langdon, Anabella Turner, Anya Mattocks, Bethany Osborne, Charli Grimes, Claire Mitchell, David Adegbite, Emma Bridgeman, Emma Scott, Fiona Perkins, Francesca Bayley, Gabriella Ruffino, Gabriella Valentine, Grace Tilzey, James Campling, Johanna Kellett Wright, Julia Brzezinska, Julie Cloake, Katarina Milutinovic, Kate Helliker, Katie Maughan, Kazminder Fox, Konstantina Minou, Lana Ward, Leah Fleming, Leigh Morrison, Lily Smart, Louise Wright, Lucy Grimwood, Maddalena Bellavia, Marianne Vasquez, Maria Garcia Gonzalez, Milo Jeenes-Flanagan, Natalie Chang, Niall Grace, Nicola Manning, Oliver Griffiths, Pip Croxford, Peter Sequenza, Rajeka Lazarus, Rhian Walters, Robin Marlow, Robyn Heath, Rupert Antico, Sandi Nammuni Arachchge, Seevakumar Suppiah, Taslima Mona, Tawassal Riaz, Vicki Mackay, Zandile Maseko, Zoe Taylor, Zsolt Friedrich, Zsuzsa Szasz-Benczur

**Affiliations:** 1 Population Health Sciences, University of Bristol, Bristol, UK; 2 NIHR Health Protection Research Unit in Behavioural Science and Evaluation, University of Bristol, Bristol, UK; 3 Dept of Engineering Mathematics, University of Bristol, Bristol, UK; 4 Clinical Research and Imaging Centre, UHBW NHS Trust, Bristol, UK; 5 Bristol Vaccine Centre, University of Bristol, Bristol, UK; 6 Academic Respiratory Unit, Southmead Hospital, University of Bristol, Bristol, UK; 7 Cellular & Molecular Medicine, University of Bristol, Bristol, UK; 8 North Bristol NHS Trust, Southmead Hospital, Bristol, UK

**Keywords:** Respiratory tract infections, Pneumonia, Public health surveillance, Missed diagnosis, Age factors

## Abstract

**Background:**

Lower Respiratory Tract Infections (LRTI) pose a serious threat to older adults but may be underdiagnosed due to atypical presentations. Here we assess LRTI symptom profiles and syndromic (symptom-based) case ascertainment in older (≥ 65y) as compared to younger adults (< 65y).

**Methods:**

We included adults (≥ 18y) with confirmed LRTI admitted to two acute care Trusts in Bristol, UK from 1st August 2020- 31st July 2022. Logistic regression was used to assess whether age ≥ 65y reduced the probability of meeting syndromic LRTI case definitions, using patients’ symptoms at admission. We also calculated relative symptom frequencies (log-odds ratios) and evaluated how symptoms were clustered across different age groups.

**Results:**

Of 17,620 clinically confirmed LRTI cases, 8,487 (48.1%) had symptoms meeting the case definition. Compared to those not meeting the definition these cases were younger, had less severe illness and were less likely to have received a SARS-CoV-2 vaccination or to have active SARS-CoV-2 infection. Prevalence of dementia/cognitive impairment and levels of comorbidity were lower in this group.

After controlling for sex, dementia and comorbidities, age ≥ 65y significantly reduced the probability of meeting the case definition (aOR = 0.67, 95% CI:0.63–0.71). Cases aged ≥ 65y were less likely to present with fever and LRTI-specific symptoms (e.g., pleurisy, sputum) than younger cases, and those aged ≥ 85y were characterised by lack of cough but frequent confusion and falls.

**Conclusions:**

LRTI symptom profiles changed considerably with age in this hospitalised cohort. Standard screening protocols may fail to detect older and frailer cases of LRTI based on their symptoms.

**Supplementary Information:**

The online version contains supplementary material available at 10.1186/s12879-024-09425-7.

## Introduction

Lower Respiratory Tract Infections (LRTI) are a leading cause of morbidity and mortality and accounted for more than 2 million deaths per year before the SARS-CoV-2 virus emerged [1]. The SARS-CoV-2 pandemic has highlighted the vulnerability of older people to LRTI, with more than 2.8 million excess deaths estimated among people aged > 70 years (y) in 2020 [2]. Although older adults suffer disproportionately high rates of LRTI [3], diagnoses may be missed as patient frailty deters clinicians from taking samples and diagnostic tests perform poorly in this group [4, 5]. This places greater importance on syndromic or symptom-based diagnoses to detect and treat cases promptly, avoid secondary transmission, and inform public health interventions to reduce disease burden [6]. 

The UK National Institute of Health and Care Excellence (NICE) advises that symptoms such as fever, cough, and increased/abnormal sputum production are indicative of LRTI [7]. However, these symptoms may not manifest in older age [5, 8]. Age can directly influence the way LRTI presents as immunosenescence results in a less robust immune response [9], and as lung function declines [3]. In addition, chronic medical conditions accumulate through life and can mask the clinical features of infection [5], while dementia and cognitive impairment are increasingly common in older adults and may prevent people from articulating their symptoms [10]. 

Older adults account for a growing proportion of the global population therefore detecting LRTI in this vulnerable group is a public health priority [10, 11]. We investigated whether syndromic case ascertainment was different in older adults compared to younger adults, by analysing LRTI symptom profiles of cases recruited to an ongoing prospective cohort study in Bristol, UK.

## Methods

### Ethics

The study was approved by the Health Research Authority Research Ethics Committee East of England, Essex, reference 20/EE/0157. Informed consent was obtained from cognisant patients, and declarations for participation from consultees for individuals lacking capacity. Patients who declined consent were not included in this analysis. For individuals for whom an approach to seek consent could not be made, data were included with approval from the Clinical Advisory Group (20/CAG/0138).

### Study population

Adults (≥ 18y) admitted to both acute care hospitals in Bristol, UK, were recruited to the AvonCAP study (ISRCTN:17,354,061, registered 03/03/2021) if they had a clinical or radiological diagnosis of acute lower respiratory tract disease (aLRTD), or if they showed > 2 signs or symptoms of LRTD on admission (Supplementary data 1). The study protocol is published containing full recruitment details [12]. Patients recruited to the AvonCAP study were followed up for 30 days and those with a final diagnosis of lower respiratory tract infection (LRTI) who were hospitalised between 1st August 2020 and 31st July 2022 were included in this analysis.

Confirmed LRTI was defined as evidence of active infection plus aLRTD, or a clinician diagnosis of LRTI, or a positive laboratory or radiological test for respiratory infection (Supplementary data 1). LRTI were classed as pneumonia if there were confirmed radiological changes compatible with infection, or when the treating clinician diagnosed pneumonia [13]. SARS-CoV-2 infection was defined as lower respiratory tract disease and a positive test result for SARS-CoV-2 on/during hospitalisation or within 7-days prior to hospital admission, using the established UK Health Security Agency (UKHSA) diagnostic assay deployed at the time.

### Data

Demographic and clinical data were systematically collected from patient records using REDCap [14]. They included a Rockwood clinical frailty score (a score > 4 indicates frailty), ranging from people who need help with higher order instrumental activities of daily living (IADL) to those who are completely dependent [15]. Comorbidity was assessed using the Charlson Comorbidity Index (CCI) [16] excluding scores assigned for a diagnosis of dementia (which were recorded separately), with CCI scores grouped in to 4 categories: 0, 1–2, 3–4 and > 4. Disease severity was measured using the CRB-65 score on admission, with a point assigned for each of: acute confusion (Abbreviated Mental Test Score ≤ 7); raised respiratory rate (≥ 30) and low blood pressure (systolic < 90mmHg or diastolic ≤ 60mmHg). As no points were assigned for age ≥ 65y since this was our outcome of interest, we term this the CRB score.

The results of standard-of-care laboratory (virological and/or bacteriological) tests, chest radiology and clinical findings were also recorded. A respiratory physician reviewed all patient records, including clinician notes and investigation results, to validate the final clinical diagnosis according to pre-specified diagnostic criteria (Supplementary data 1).

To test our hypothesis that LRTI is less likely to be identified among older as compared to younger adults, we constructed a syndromic case definition for suspected LRTI using a list of signs and symptoms published in guidance from the National Health Service (NHS), British Medical Journal (BMJ), and NICE (Supplementary data 2) [17, 18]. To meet this case definition, patients had to present with cough plus fever (reported fever/chills or temperature > 38.0 °C or < 35 °C), or with at least three of the following: cough, fever, breathlessness, wheeze, pleurisy (chest pain on breathing), abnormal sputum production, myalgia, headache, or general deterioration (weakness/fatigue/anorexia).

### Study objectives

The primary objective was to determine whether older adults hospitalised with LRTI were less likely to meet the syndromic LRTI case definition when compared to younger adults. Secondary objectives were: (a) describe the cohort of adults with LRTI by factors that influence clinical presentation (age, sex, levels of comorbidity/frailty, and diagnosis of dementia or cognitive impairment), and (b) assess which symptom profiles characterised older adults.

### Statistical analysis

Categorical data were summarised as counts and percentages, continuous data as medians with interquartile (IQR) ranges. The characteristics of patient groups were compared using Fisher exact tests for dichotomous variables, two-sided Kolmogorov-Smirnov tests for continuous variables and Wilcoxon rank sum tests for score variables. Density plots were used to visualise changes in cases’ age distribution and SARS-CoV-2 positivity over time, to better understand sources of bias.

To assess whether LRTI case ascertainment was lower in adults ≥ 65y compared to those < 65y we built a multivariable logistic regression model. The primary exposure was age ≥ 65y, and the outcome was meeting the syndromic LRTI case definition at presentation (Yes/No). Covariates were selected based on our understanding of relationships between age, LRTI symptom expression and associated factors (Supplementary data 4–5), along with results of our descriptive analyses (Table 1). In case the study population was biased towards people presenting with ‘classical’ symptoms of LRTI (see study inclusion criteria, Supplementary data 1) we conducted a sensitivity analysis restricted to cases recruited with a clinical or radiological diagnosis of LRTD, *and* a final diagnosis of radiologically- confirmed community- acquired pneumonia (CAP).

**Table 1:** Characteristics of LRTI cases by whether presenting symptoms meet the case definition

Characteristic	Not meeting LRTI case definition^a^ *N* = 9,133	Meeting LRTI case definition^a^ *N* = 8,487	*p*-value^b^
Age (yrs) at admission	75 (58, 85)	68 (52, 80)	< 0.001
Age group			< 0.001
18–24	189 (2.1%)	254 (3.0%)	
25–34	506 (5.5%)	492 (5.8%)	
35–44	549 (6.0%)	691 (8.1%)	
45–54	681 (7.5%)	958 (11%)	
55–64	1,051 (12%)	1,327 (16%)	
65–74	1,486 (16%)	1,641 (19%)	
75–84	2,301 (25%)	1,851 (22%)	
>84	2,370 (26%)	1,273 (15%)	
SARS-CoV-2 test positive	4,259 (47%)	3,051 (36%)	< 0.001
Vaccinated against SARS-CoV-2	5,524 (65%)	4,865 (61%)	< 0.001
Unknown	683 (7.5%)	508 (6.0%)	
CRB score^c^			< 0.001
0	5,853 (64%)	5,857 (69%)	
1	2,731 (30%)	2,269 (27%)	
2	501 (5.5%)	332 (3.9%)	
3	41 (0.4%)	25 (0.3%)	
Unknown	7 (0.08%)	4 (0.05%)	
Male sex	4,601 (50%)	4,353 (51%)	0.2
Care home resident	910 (10.0%)	482 (5.7%)	< 0.001
Dementia/ cognitive impairment	1,285 (14%)	668 (7.9%)	< 0.001
Clinically frail^d^	3,860 (55%)	2,785 (39%)	< 0.001
Unknown	2,145 (23%)	1,329 (16%)	
Comorbidity score^e^			0.043
0	3,640 (40%)	3,449 (41%)	
1–2	3,707 (41%)	3,503 (41%)	
3–4	1,245 (14%)	1,137 (13%)	
>4	534 (5.9%)	394 (4.6%)	
Unknown	7 (0.08%)	4 (0.05%)	

^a^Median (IQR); n (%)

^b^Welch Two Sample t-test; Wilcoxon rank sum test; Fisher’s Exact Test for Count Data

^c^Pneumonia severity score, 1 point assigned for each of: acute confusion, raised respiratory rate, low blood pressure

^d^Rockwood frailty score > 4

^e^Using a modified Charlson Comorbidity Index, minus points for age and dementia

To explore differences in symptom expression we calculated the posterior log-odds of each symptom being expressed in adults ≥ 65y versus adults < 65y, employing an empirical Bayesian approach with a multinomial model and an informative Dirichlet prior estimated from the data. To account for greater variance in estimates for rarer symptoms, log-odds were weighted according to the frequency of each symptom observed and presented as z-scores of the log odds ratio.

To assess whether symptom profiles changed with age, we generated a matrix of symptom frequencies by 10y age bands and produced a heatmap to illustrate how common each symptom was relative to other symptoms within each age band. We applied a dendrogram to the heatmap, using a hierarchical clustering function based on a Euclidean distance matrix, showing how age bands were related in terms of symptom expression [19]. Next, we conducted Principal Component Analysis (PCA) to identify linear combinations of symptoms (Principal Components, [PC]), such that each PC contributed to overall variance between age bands but was uncorrelated with other PCs [20]. We then selected PCs, each representing a different symptom profile, that explained most of the variation and applied a k-means clustering algorithm to identify which 10y age bands were most closely related in terms of PCs/ symptom profiles [21]. We used silhouette scores to determine how many clusters to create (Supplementary Data 7) [22], and age bands were assigned to clusters such that the sum of squared distances between age bands and cluster centroid was minimised. Finally, k-means clusters were presented in a graph with age bands positioned according to their PC values, such that age bands with similar symptom profiles appeared close together and those that differed were further apart.

All analyses were conducted using R statistical software version 4.2.1 [23]. Missing data were limited to CRB score and comorbidity level variables - each accounting for 0.06% of the sample. No imputation was performed and multivariable logistic regression analyses only included participants with complete data. Statistical significance was defined using a 2-sided significance level of α = 0∙05.

## Results

### Descriptive analysis

Overall, 21,447 adults were hospitalised with suspected aLRTD, of which 18,680 (87.1%) had ≥ 2 signs or symptoms according to recruitment criteria (Supplementary data 1). Of these, 20,524 (95.7%) were tested for respiratory bacteria and/ or viruses, and 20,194 (94.2%) underwent radiological investigation during their admission. A total of 17,620 (82.0%) were diagnosed with LRTI including 7,310 (41.5%) with a positive SARS-CoV-2 test and 10,125 (57.5%) with pneumonia (of which 90.4% had radiological confirmation). Among confirmed LRTI cases, 8,487 (48.1%) had symptoms that met the syndromic LRTI case definition (Supplementary data 3). These cases were younger, less likely to live in a care home and less likely to have received the SARS-CoV-2 vaccine or have active SARS-CoV-2 infection. They were also less likely to have a comorbidity score of CCI > 4, a diagnosis of dementia/cognitive impairment, or at least one CRB score indicator of severe disease on admission as compared to cases whose symptoms did not meet the syndromic LRTI case definition (Table 1).

**Fig. 1:**
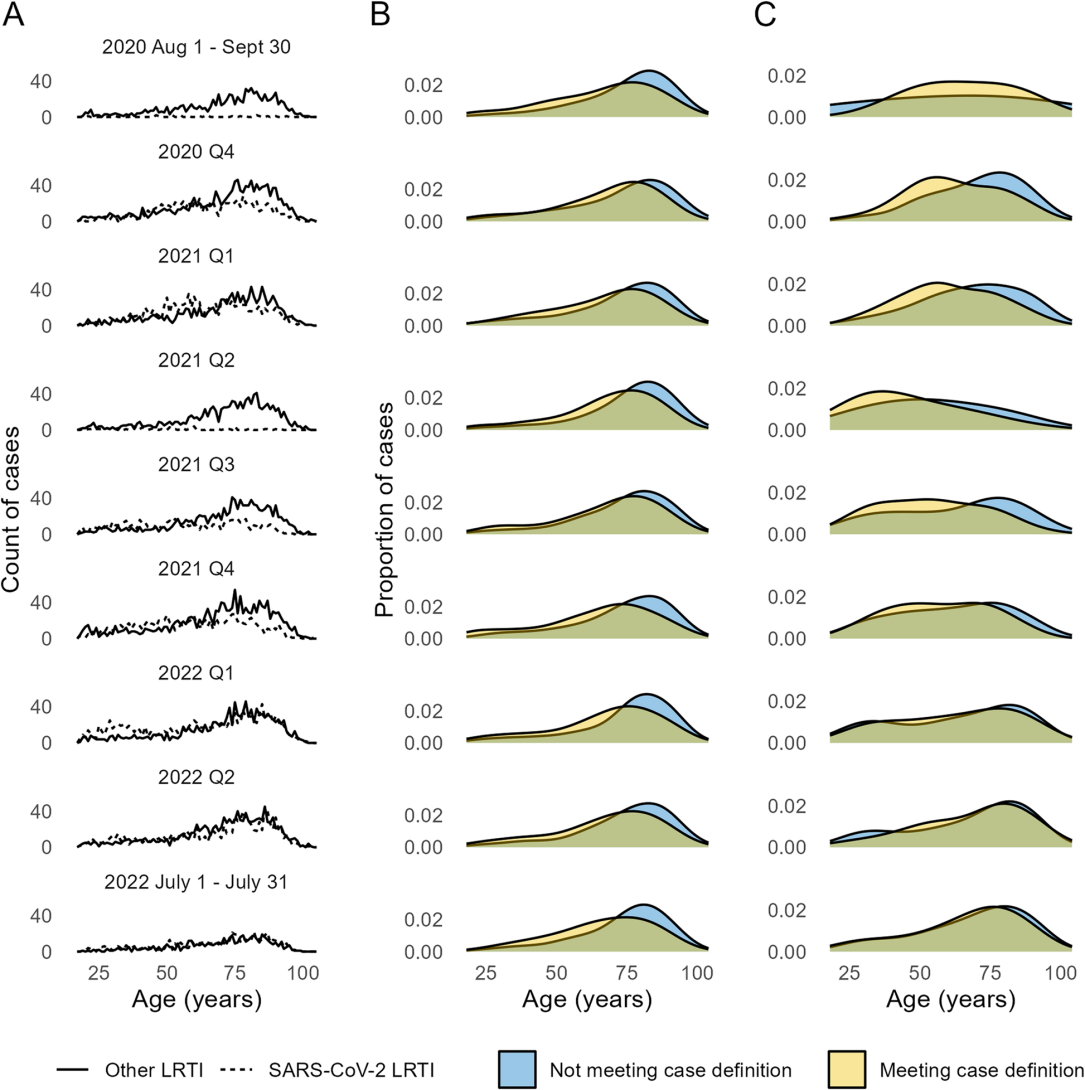
Age distribution of LRTI cases. The age distribution of LRTI cases by SARS-CoV-2 status and whether patients presented with symptom profiles consistent with the LRTI case definition. **A** Counts of cases per quarter, by year of age, with dotted lines showing counts of SARS-CoV-2 LRTI and solid lines showing counts of other LRTI; **B** Age distribution of SARS-CoV-2 negative cases and **C** SARS-CoV-2 positive cases. Each row represents a quarter (Q) from Q3 2020 through to Q3 2022, except for the first and last rows which are truncated due to the time period of this analysis

The age distribution of SARS-CoV-2 positive cases changed over time, with a peak emerging in late 2020 through 2021 which represented younger cases with symptoms largely meeting the syndromic LRTI case definition. There was also a peak in older cases, most of whom did not have symptoms meeting the case definition, which was consistent except in quarter 2 of 2021 (Fig. 1C). From mid-2021 older cases began to dominate, eventually mirroring the age distribution for non-SARS-CoV-2 LRTI (Fig. 1B).

### Logistic regression

The probability of symptoms meeting the syndromic LRTI case definition was significantly lower for adults aged ≥ 65y as compared to adults aged < 65y both on univariable analysis (OR = 0.62, 95% Confidence Interval (CI) 0.58–0.66) and after adjusting for sex, presence of dementia/ cognitive impairment, and CCI scores of > 4 (aOR = 0.67, 95%CI 0.63–0.71; Table 2, model 1a). Dementia and comorbidities (CCI score) were included as confounding variables in the model however these factors can also moderate the effect of age on symptom profile (see DAG, Supplementary data 4). For comparison, we ran model 1 without these two variables and found that older age further reduced the odds of meeting the syndromic case definition (aOR = 0.62, 95%CI 0.58–0.66; Table 2, model 1b).

**Table 2:** Logistic Regression – odds of symptoms meeting LRTI case definition

	Unadjusted odds				Adjusted odds, model 1a			Adjusted odds, model 1b		
Characteristic	*N*	OR^a^	95% CI^a^	*p*-value	OR^a^	95% CI^a^	*p*-value	OR^a^	95% CI^a^	*p*-value
Aged > = 65y	17,620	0.62	0.58, 0.66	< 0.001	0.67	0.63, 0.71	< 0.001	0.62	0.58, 0.66	< 0.001
Male sex	17,620	1.04	0.98, 1.10	0.2	-	-	-	-	-	-
Dementia	17,620	0.52	0.47, 0.58	< 0.001	-	-	-			
CCI score > 4^b^	17,609	0.78	0.69, 0.90	< 0.001	-	-	-			

Model 1b excludes dementia/ cognitive impairment and CCI score. Adjusted odds are not reported for covariates as we have not accounted for confounding of these effect estimates so results may be misleading [24]

^a^*OR* Odds Ratio, *CI* Confidence Interval

^b^Modified Charlson Comorbidity Index, minus points for age and dementia

Repeating the analysis on a subgroup of patients who were not recruited based on their symptom profile, and who had a final diagnosis of radiologically- confirmed CAP (*n* = 7,193) showed that patients whose symptoms met the syndromic LRTI case definition were again younger with fewer comorbidities and lower CRB scores. In contrast to the original analysis, however, these cases were more likely to have tested positive for SARS-CoV-2 infection as compared to those whose symptoms did not meet the syndromic case definition (Supplementary data 9). In the CAP subgroup, age ≥ 65y was associated with an even lower probability of meeting the syndromic case definition as compared to age < 65y (aOR 0.53, 95%CI 0.48–0.59 after adjusting for sex, comorbidities, and dementia; Supplementary data 9).

### Symptom profiles

**Fig. 2:**
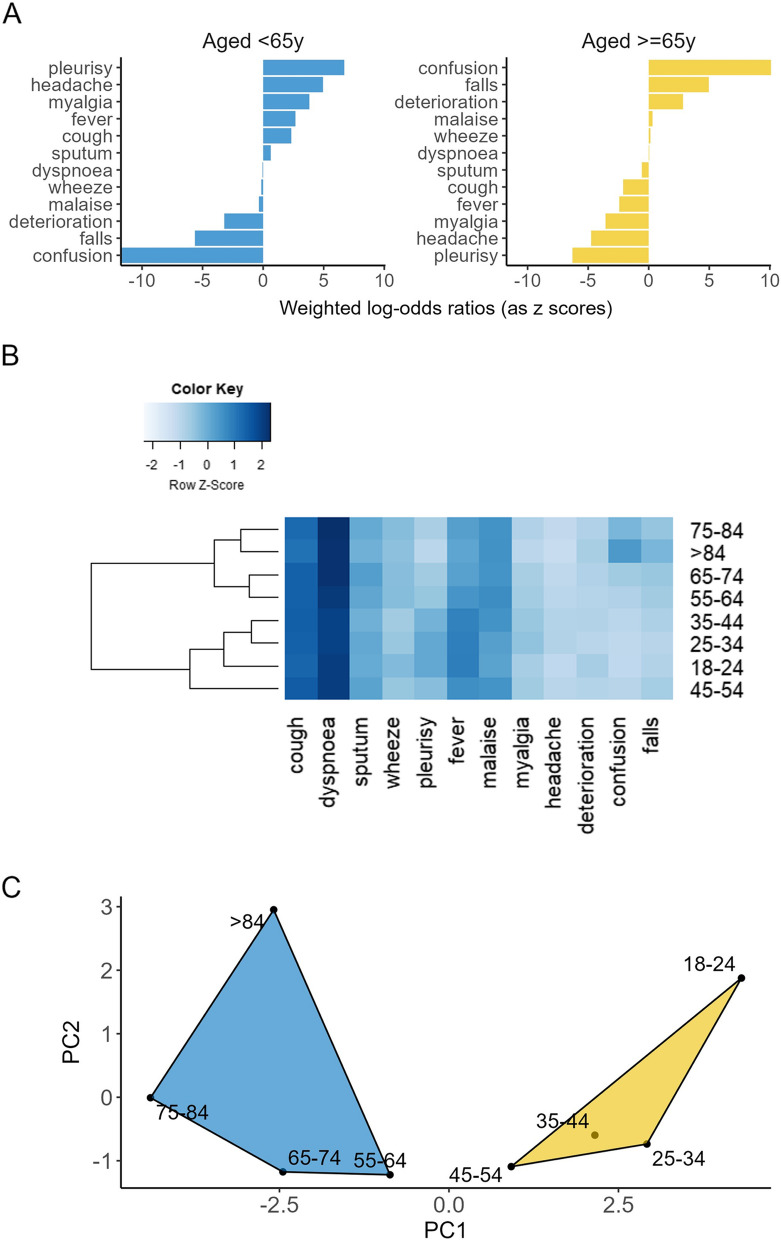
Age-specific symptom profiling for LRTI. **A** Comparison of LRTI symptom expression in older (≥ 65y) vs. younger (< 65y) adults is shown as a probability of each symptom occurring, with weighted log-odds ratios expressed as z-scores (number of standard deviations from the mean). **B** Cases are grouped in 10y age bands (rows), and cells are coloured based on frequency of a symptom within each age band relative to all other symptoms within the age band. Frequencies are presented as z-scores. **C** Age bands are assigned to clusters based on similarity of LRTI symptom profiles (Principal Components) and plotted according to PC1 and PC2 scores

Older adults (≥ 65y) were more likely to present with confusion, falls, and general deterioration and less likely to present with pleurisy, headache, cough, and sputum than younger adults (< 65y) (log-odds of symptom expression, Fig. 2A). Analysis of symptom profiles by 10y age bands showed that cough and breathlessness were the most frequent presenting symptoms across the age spectrum, except for cases ≥ 85y who were less likely to cough and more likely to experience confusion and falls (Fig. 2B).

### Cluster analysis

The heatmap and dendrogram revealed two distinct hierarchical clusters separating cases above and below 55y, with fever and pleurisy more prominent below 55y. Principal Component Analysis (PCA) of symptom frequencies by age group found that two components/ symptom profiles explained over 98% of variation between age groups. (Supplementary Data 6). Dividing the age bands into 2 k- means clusters based on PCA results (Supplementary Data 7) showed that the youngest (18-24y) and oldest (≥ 85y) age bands were most distinctive in terms of symptom profile (Fig. 2C).

## Discussion

Less than half of adults hospitalised with LRTI presented with symptoms matching the syndromic LRTI case definition. Our results suggest that older and frailer adults, particularly those with cognitive impairment or multiple comorbidities, are at greatest risk of missed diagnosis due to atypical presentations. We find that cases aged ≥ 65y, who made up almost two thirds of the cohort, were less likely to present with classical LRTI symptoms such as cough, fever and pleurisy when compared to younger cases, and that those aged ≥ 85y commonly presented with confusion and falls. This has implications for older patients whose infections may not be diagnosed and treated, as well as research and policy since standard screening protocols may underestimate disease burden and vaccine effectiveness in older age groups. It also highlights the importance of microbiological and radiological investigations in the diagnosis of LRTI.

The AvonCAP prospective cohort study provided clinically validated LRTI diagnoses and comprehensive symptoms data with which we could assess the performance of syndromic case definitions. Few studies have explored how symptom profiles evolve with age. This analysis provides further evidence that symptoms are unreliable predictors of LRTI in older patients, who are more likely to present with non- specific signs of deterioration. The diagnostic value of syndromic case definitions is therefore limited in this age group, and there should be a low threshold for laboratory/ radiological investigations to identify and treat cases of LRTI in this population.

Other studies of hospitalised adults have reported low sensitivity for LRTI case definitions in older adults; [25] in a study of veterans aged > 40y with bacterial pneumonia, younger adults (< 65y) were significantly more likely to present with breathlessness, sputum production and pleurisy than older adults (≥ 65y) [26]. Confusion and falls were common among veteran cases aged > 80y, which was attributed to high rates of dementia, but contrary to our findings, cough was as prevalent in this age group as in others [26]. Studies focussing on hospitalised adults aged > 80y have reported much higher prevalence of altered mental state (53–77%) and lower prevalence of cough (40–63%) in nursing home-acquired pneumonias as compared to community-acquired pneumonias (altered mental state: 12–45%, cough: 49–81%) [3]. This symptom profile may therefore be characteristic of frailer cases (frailty defined as a degree of dependence for daily activities of living) [15], since cases aged ≥ 85y accounted for the greatest proportion (17%) of care home residents in our cohort.

With respect to SARS-CoV-2 LRTI, which made up over 40% of our sample, data from the UK Coronavirus (COVID-19) Infection Survey also show that the probability of reporting fever declines from around the age of 60y [27]. Although fever is considered a cardinal symptom of LRTI it is a poor predictor of infection in older adults [28, 29], and its absence will contribute to case under-ascertainment.

The SARS-CoV-2 pandemic was a very unusual period and may have biased our results for several reasons. Firstly, our case mix and symptom profiles may have shifted as the result of unseasonal fluctuations in respiratory illnesses, the emergence of new SARS-CoV-2 variants, and changes in hospital admission thresholds for care home residents and other vulnerable groups [30, 31]. We saw a bimodal age distribution of SARS-CoV-2 positive cases from the end of 2020 through to 2022 which probably reflects the emergence of new variants Alpha and Delta while older adults were prioritised for vaccination. This resulted in a greater proportion of hospitalised cases among younger adults [27, 32], whilst the steady peak in older aged cases represents frail individuals with a low threshold for hospital admission. The age distribution of sexes also differed for SARS-CoV-2 when compared to other forms of LRTI (Supplementary data 10), with a peak in young females possibly representing pregnant women. Assuming these individuals had a lower severity threshold for admission, they would have been less likely to express symptoms and meet the LRTI case definition. Secondly, the SARS-CoV-2 virus was prioritised for testing above other pathogens during the study period, and symptoms may have differed from other causes of LRTI (Supplementary Data 11), nonetheless the majority of LRTI in our cohort and a previous AvonCAP cohort were SARS-CoV-2 negative [32]. Thirdly, older adults were prioritised for SARS-CoV-2 vaccination, which reduced severity of illness and therefore symptom expression and may have enhanced the effect of older age on probability of meeting the case definition [33]. Finally, a series of lockdowns prevented all but the most severe cases of infection from presenting to hospital. Restrictions were particularly tight for ‘clinically vulnerable’ individuals so that this population, around 40% of whom were aged < 60y, and who may have had comorbidities without necessarily being frail, were less likely to be included in this study [34]. 

The fact that our population of hospital patients is skewed towards individuals who either had severe illness or were frail is a major limitation. By excluding milder (and possibly less symptomatic) cases of LRTI occurring in young, fit individuals we may be overestimating the differences in symptom profiles between younger and older adults, although in this study population clinical risk on admission was not noticeably higher in younger age groups (Supplementary Data 8).

Our analysis approach also limits the interpretation of results, largely because we relied on our own syndromic case definition to assess whether older adults with LRTI are less likely to be diagnosed at presentation. We feel that the case definition is representative of what clinicians will look for in a suspected case of LRTI, based on reputable sources, however this is not an evaluation of a clinical diagnostic tool. Additionally, the regression model did not provide an estimate of the total effects of age on symptom profile since it included dementia and comorbidity scores as confounders (see DAG in Supplementary Data 4). Outputs from a model without these covariates (Table 2, model 1b) suggest a strong total effect of age on symptom profile but may be confounded. Finally, data were gathered from hospital case notes so misclassification of clinical syndromes and diagnoses is possible, and our cohort may not be representative of all hospitalised LRTI. However, enrolment criteria were broad and case review thorough so that we are confident that the risk of misclassification was low.

In conclusion, this analysis provides further evidence that older adults with LRTI present atypically, reducing the likelihood of timely diagnosis and successful intervention. Syndromic case definitions are less useful to identify older cases with non- specific symptoms so microbiological or radiological confirmation of LRTI is especially important in this vulnerable population. Further work is needed to determine symptom profiles in non-hospitalised cases of LRTI, including those with cognitive impairment and living in long-term care.

## Supplementary Information

Supplementary Material 1.

Supplementary Material 2.

Supplementary Material 3.

Supplementary Material 4.

Supplementary Material 5.

Supplementary Material 6.

Supplementary Material 7.

Supplementary Material 8.

Supplementary Material 9.

Supplementary Material 10.

## Data Availability

The raw datasets analysed during the current study are not publicly available to preserve the confidentiality of our participants. The code used for this analysis is available on GitHub: https://github.com/bristol-vaccine-centre/LRTI_symptoms.
