# Supplementary material for: Syndromic case definitions for lower respiratory tract infection (LRTI) are less sensitive in older age: an analysis of symptoms among hospitalised adults
Source: BMC Infect Dis. 2024 Jun 7;24:568. doi: 10.1186/s12879-024-09425-7 (PMC11157799; doi:10.1186/s12879-024-09425-7)
Supplement: Supplementary file 1 — Supplementary Material 1. [file 12879_2024_9425_MOESM1_ESM.docx]

**SUPPLEMENTARY DATA 1: Study population inclusion and exclusion criteria.**

**Figure S1**: Flowchart illustrating how patients were included in this study.

Recruit patients with suspected aLRTD

Select cases with a final diagnosis of LRTI

Follow-up 30 days

| 1. **AvonCAP study inclusion criteria** | 1. **Criteria for final diagnosis of LRTI** |
| --- | --- |
| **INCLUSION**   1. Clinical or radiologic diagnosis of pneumonia or an acute LRTD, **OR** 2. ≥2 of the following signs/ symptoms: fever (>38.0°C) or hypothermia (< 35.5°C), pleuritic chest pain, cough, sputum production/ purulence, dyspnoea, tachypnoea (≥20 breaths/ minute), abnormal auscultatory findings, radiologic findings consistent with LRTD.   **EXCLUSION**   1. Age <18 years. 2. Signs and symptoms of LRTD manifest after being hospitalized for ≥48 hours (either at current hospital, another transferring hospital, or a combination of these), unless admitted with current, previously proven, or suspected COVID-19 infection. 3. Previous enrolment in the study and within 7 days of discharge, unless admitted with current, previously proven, or suspected COVID-19 infection. 4. LRTD-related diagnosis excluded at the time of enrolment, or alternative diagnosis confirmed. | 1. Clinical records contain a diagnosis or treatment consistent with LRTI, **OR** 2. Positive laboratory test for pneumococcus, RSV, SARS-CoV-2, or any other infectious respiratory pathogen, **OR** 3. Evidence of both: 4. Active infection, i.e. at least 1 of the following conditions: reported fever, reported chills, measured temperature of >38.0°C or <35°C, or an abnormal white blood cell count or differential), **AND** 5. Lower respiratory tract disease (at least 1 of the following conditions: abnormal breath sounds, documented tachypnea, cough, sputum production, or dyspnoea). |
| aLRTD: acute Lower Respiratory Tract Disease; LRTI: Lower Respiratory Tract Infection. | |

**SUPPLEMENTARY DATA 2: Diagnostic symptoms for Lower Respiratory Tract Infection.**

**Table S2**: Comparison of symptom profiles listed by the National Health Service (NHS), British Medical Journal (BMJ), and National Institute for Health and Care Excellence (NICE).

|  | **NHS public site** ^[[1]](#footnote-2)^ | **BMJ Best Practice** ^[[2]](#footnote-3),^^[[3]](#footnote-4)^ | **NICE guidelines ^d^** |
| --- | --- | --- | --- |
| Fever |  |  |  |
| Cough |  |  |  |
| Breathlessness |  |  |  |
| Wheeze |  |  |  |
| Pleuritic chest pain |  |  |  |
| Abnormal sputum |  |  |  |
| Myalgia |  |  |  |
| Headache |  |  |  |
| Tiredness/ fatigue |  |  |  |
| Malaise |  |  |  |
| Altered sense of taste/ smell |  |  |  |
| NHS = UK National Health Service; BMJ = British Medical Journal; NICE = National Institute for Health & Care Excellence.  = General features of LRTI. = Common features of SARS-CoV-2 infection. | | | |
|  | | |  |

**SUPPLEMENTARY DATA 3: Study flow diagram.**

**Figure S3**: Flowchart illustrating progress from study recruitment to inclusion in this analysis.


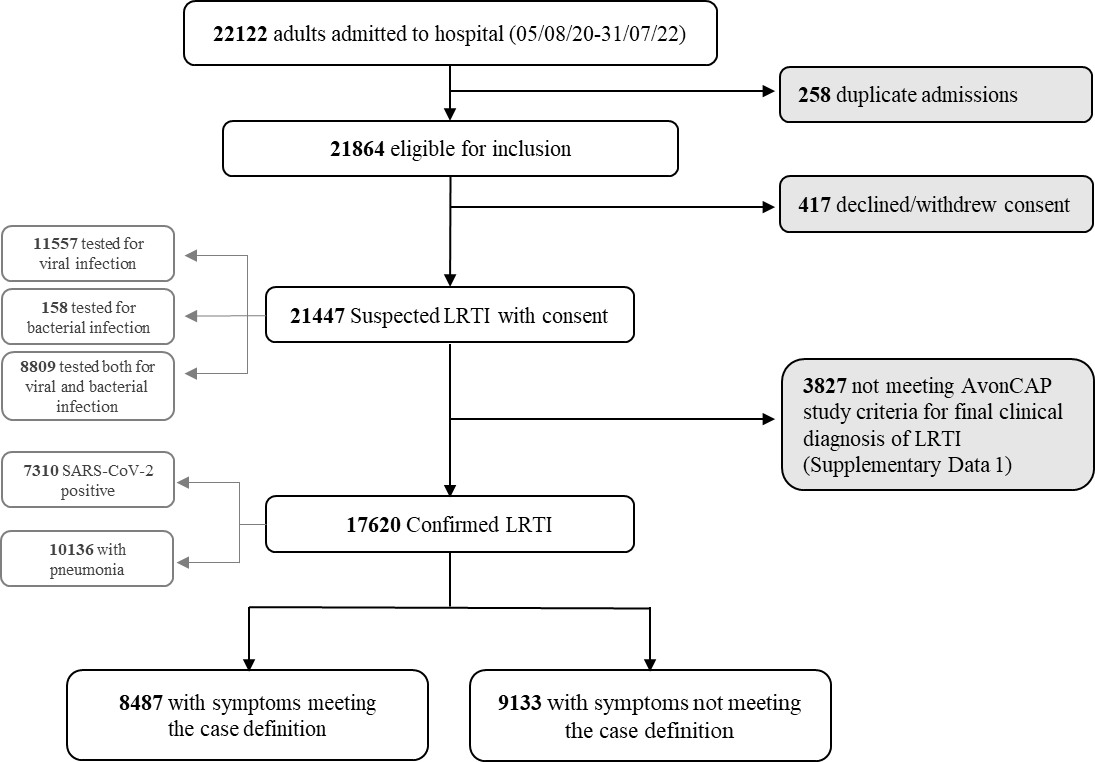


**SUPPLEMENTARY DATA 4: Directed Acyclic Graph (DAG).**


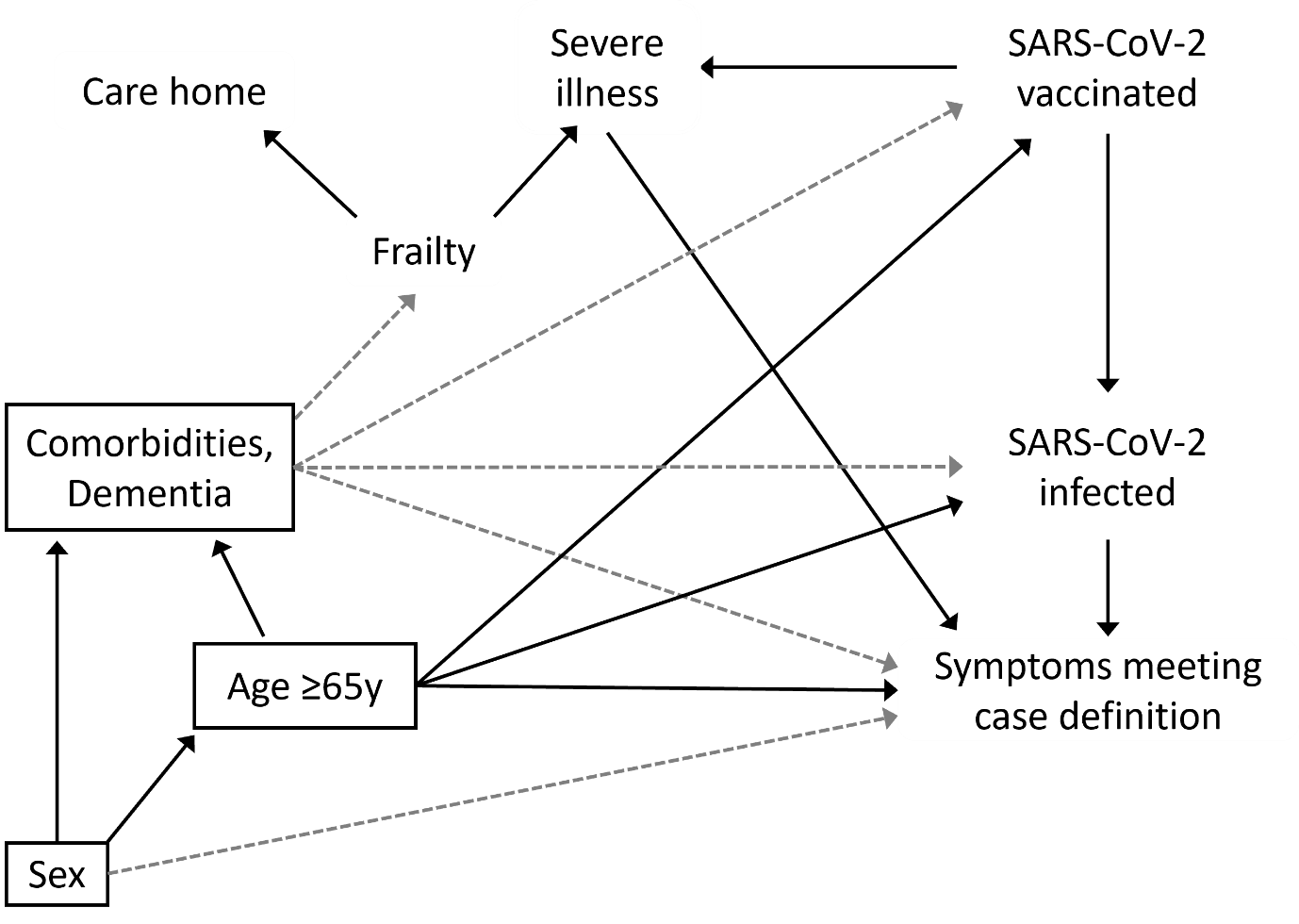


**Figure S4**: Directed Acyclic Graph illustrating causal pathways between the exposure of interest (age ≥65 years) and the outcome of interest (symptoms meeting the LRTI case definition). Boxes indicate variables that are included in regression model 1. Solid black arrows indicate pathways by which the EOI (age ≥65y) affects the outcome (symptoms meeting case definition). Grey dashed arrows indicate pathways that are blocked by controlling for sex and the presence of comorbidities/ dementia.

**SUPPLEMENTARY DATA 5: Cross- correlation matrix.**

**Figure S5**: Matrix showing pairwise correlations between explanatory variables and the outcome of interest (symptoms meeting the LRTI case definition).


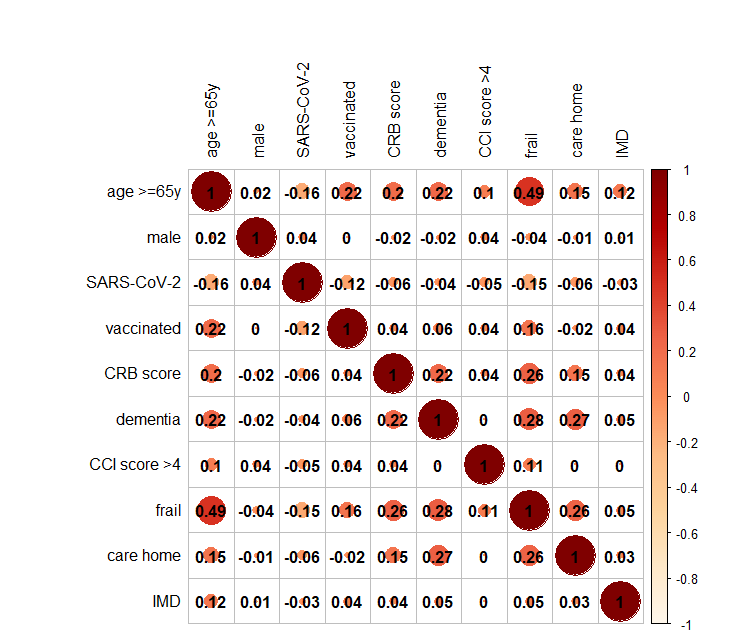


**SUPPLEMENTARY DATA 6: Principal Component Analysis (PCA)**

The first step in our clustering analysis was to distil the symptoms dataset into symptom profiles that characterised the differences between 10- year age bands. Variables included in the PCA were: 10-year age band (a categorical variable with 8 levels), plus: cough, dyspnoea, sputum, wheeze, pleurisy, fever, malaise, myalgia, headache, deterioration, confusion, and falls (all expressed as frequencies).

Together, PC1 and PC2 explained 98% of symptom variation between age bands (see table below).

**Table S6**: Variation between age bands explained by each Principal Component

|  | **Principal component** | | | | | | | |
| --- | --- | --- | --- | --- | --- | --- | --- | --- |
|  | **PC1** | **PC2** | **PC3** | **PC4** | **PC5** | **PC6** | **PC7** | **PC8** |
| Standard deviation | 3.06 | 1.57 | 0.38 | 0.20 | 0.06 | 0.06 | 0.04 | 0.00 |
| Proportion of variance explained | 0.78 | 0.20 | 0.01 | 0.00 | 0.00 | 0.00 | 0.00 | 0.00 |
| Cumulative proportion of variance | 0.78 | 0.98 | 1 | 1 | 1 | 1 | 1 | 1 |

**Figure S6**: Bar plot showing the relative contribution of each symptom variable to Principal Components 1 and 2. Principal Component 1 (PC1) was largely defined by the following symptoms: breathlessness, malaise, fever, cough, wheeze, and sputum production. Headache and pleurisy contributed the most to Principal Component 2 (PC2).

**
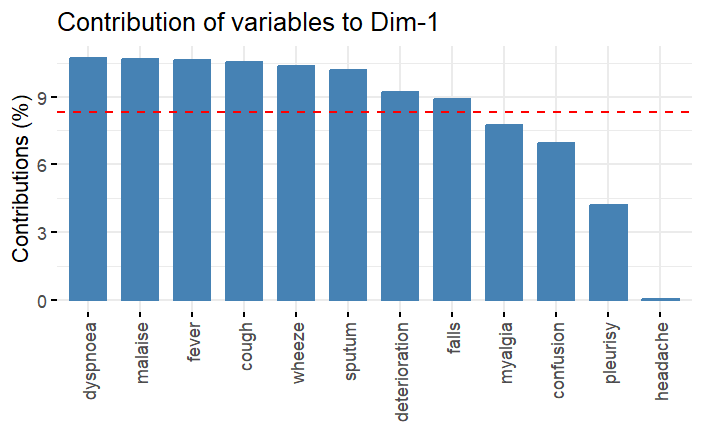

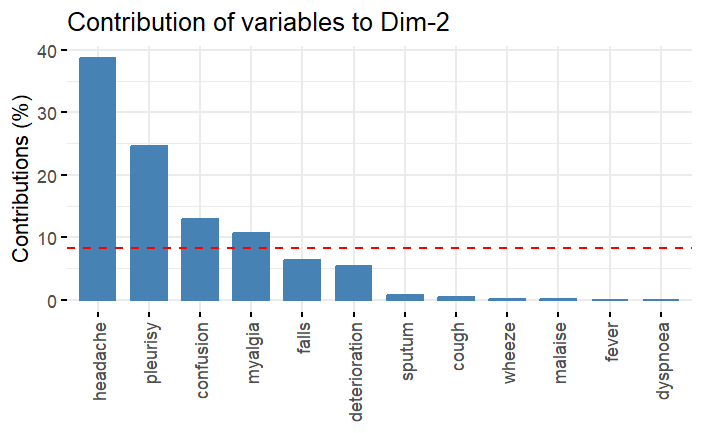
**

**SUPPLEMENTARY DATA 7: Cluster identification**

Having reduced the symptoms data into Principal Components that explained almost all variation between 10- year age bands, we wanted to assess how age bands were clustered.

**Determining the optimal number of clusters (k)**

**Figure S7A**: Plot showing the average silhouette width (a measure of how similar values within a cluster are to each other, compared to values in other clusters), by the number of clusters. A silhouette width closer to 1 indicates that clusters are well differentiated, and a silhouette width closer to -1 indicates that clusters are poorly differentiated. This plot indicates that k = 2 gives the best overall alignment with cluster centroids, with an average silhouette width of around 0.45.


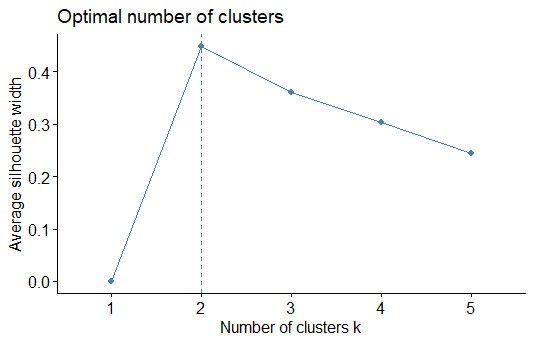


**Assigning clusters**

Once the number of clusters (k=2) was determined, the kmeans algorithm selected two data points at random to form a cluster of size 1. Every data point was then assigned to the closest cluster, and a centroid calculated as the central point of that cluster. The process was repeated until the clusters became stationary.

**SUPPLEMENTARY DATA 8: AvonCAP study population (n = 21,447)**

**Clinical investigations**

**Table S8A**: Investigations during admission, by number of signs/ symptoms of aLRTD on admission.

| **Investigations** | **<2 signs/ symptoms**,  N = 2,754*^1^* | **>=2 signs/ symptoms**,  N = 18,680*^1^* |  |
| --- | --- | --- | --- |
| Lab test only | 786 (29%) | 346 (1.9%) |  |
| Radiology only | 67 (2.5%) | 736 (4.0%) |  |
| Lab and radiology | 1,865 (69%) | 17,514 (94%) |  |
| No lab/ radiology | 0 (0%) | 0 (0%) |  |
| Unknown | 36 (1.3%) | 84 (0.5%) |  |
| *^1^* n (%) | | |  |
|  | | | |

**NEWS2 clinical risk on admission, by age group**

**Table S8B**: The National Early Warning Score (NEWS2) is used to triage patients based on their clinical condition on arrival to hospital.^[[4]](#footnote-5)^ It is based on the following physiological parameters: respiration rate, oxygenation (SpO_2_) levels, oxygen use, systolic blood pressure, pulse, levels of consciousness, and temperature. Using standardised thresholds published by the Royal College of Physicians, cases are assigned a level of clinical risk: low, low- medium, medium or high risk.

|  | **Age group** | | | | | | | |
| --- | --- | --- | --- | --- | --- | --- | --- | --- |
| **NEWS2 risk** | **18-24** N=533*^1^* | **25-34** N=1,142*^1^* | **35-44**, N=1,429*^1^* | **45-54**, N=1,896*^1^* | **55-64**, N=2,859*^1^* | **65-74**, N=3,887*^1^* | **75-84**, N=5,128*^1^* | **>84**, N=4,573*^1^* |
| Low | 327 (68%) | 723 (70%) | 812 (63%) | 980 (59%) | 1,290 (51%) | 1,683 (49%) | 2,216 (48%) | 1,865 (44%) |
| Low-medium | 128 (27%) | 238 (23%) | 349 (27%) | 507 (30%) | 942 (37%) | 1,381 (40%) | 1,969 (43%) | 2,076 (49%) |
| Medium | 20 (4.2%) | 59 (5.7%) | 97 (7.5%) | 140 (8.4%) | 228 (9.0%) | 297 (8.6%) | 307 (6.7%) | 215 (5.1%) |
| High | 5 (1.0%) | 14 (1.4%) | 31 (2.4%) | 38 (2.3%) | 72 (2.8%) | 86 (2.5%) | 100 (2.2%) | 39 (0.9%) |
| Unknown | 53 (9.9%) | 108 (9.5%) | 140 (9.8%) | 231 (12%) | 327 (11%) | 440 (11%) | 536 (10%) | 378 (8.3%) |
| *^1^* n (%) |  |  |  |  |  |  |  |  |

**SUPPLEMENTARY DATA 9: Sensitivity analysis**

**Table S9A**: Characteristics of radiologically- confirmed CAP cases by whether presenting symptoms meet the case definition.

| **Characteristic** | | **Not meeting LRTI case definition^a^** N = 3,317*^1^* | | **Meeting LRTI case definition^a^**  N = 3,876*^1^* | | **p-value^b^** | |
| --- | --- | --- | --- | --- | --- | --- | --- |
| Age (yrs) at admission | | 77 (62, 86) | | 68 (53, 80) | | <0.001 | |
| Age group | |  | |  | | <0.001 | |
| 18-24 | | 23 (0.7%) | | 50 (1.3%) | |  | |
| 25-34 | | 78 (2.4%) | | 189 (4.9%) | |  | |
| 35-44 | | 151 (4.6%) | | 323 (8.3%) | |  | |
| 45-54 | | 257 (7.7%) | | 474 (12%) | |  | |
| 55-64 | | 398 (12%) | | 659 (17%) | |  | |
| 65-74 | | 567 (17%) | | 754 (19%) | |  | |
| 75-84 | | 880 (27%) | | 809 (21%) | |  | |
| >84 | | 963 (29%) | | 618 (16%) | |  | |
| SARS-CoV-2 test positive | | 1,285 (39%) | | 1,752 (45%) | | <0.001 | |
| Vaccinated against SARS-CoV-2 | | 1,757 (57%) | | 1,940 (53%) | | 0.006 | |
| Unknown | | 208 (6.3%) | | 227 (5.9%) | |  | |
| CRB score^c^ | |  | |  | | <0.001 | |
| 0 | | 1,926 (58%) | | 2,555 (66%) | |  | |
| 1 | | 1,106 (33%) | | 1,124 (29%) | |  | |
| 2 | | 260 (7.8%) | | 177 (4.6%) | |  | |
| 3 | | 22 (0.7%) | | 17 (0.4%) | |  | |
| Unknown | | 3 (0.09%) | | 3 (0.08%) | |  | |
| Male sex | | 1,786 (54%) | | 2,150 (55%) | | 0.2 | |
| Care home resident | | 383 (12%) | | 204 (5.3%) | | <0.001 | |
| Dementia/ cognitive impairment | | 513 (15%) | | 292 (7.5%) | | <0.001 | |
| Clinically frail^d^ | | 1,593 (58%) | | 1,246 (38%) | | <0.001 | |
| Unknown | | 583 (17.6%) | | 568 (14.7%) | |  | |
| Comorbidity score^e^ | |  | |  | | <0.001 | |
| 0 | | 1,165 (35%) | | 1,616 (42%) | |  | |
| 1-2 | | 1,436 (43%) | | 1,576 (41%) | |  | |
| 3-4 | | 489 (15%) | | 490 (13%) | |  | |
| >4 | | 224 (6.8%) | | 191 (4.9%) | |  | |
| Unknown | | 3 (0.09%) | | 3 (0.08%) | |  | |
| ^a^ Median (IQR); n (%) | | | | | | | |
| ^b^ Welch Two Sample t-test; Wilcoxon rank sum test; Fisher’s Exact Test for Count Data. | | | | | | | |
| ^c^ Pneumonia severity score, 1 point assigned for each of:   acute confusion, raised respiratory rate, low blood pressure. | | | | | | | |
| ^d^ Rockwood frailty score >4. | | | | | | | |
| ^e^ Using a modified Charlson Comorbidity Index, minus points for age and dementia. | | | | | | | |

**Table S9B**:.Logistic regression – odds of symptoms meeting LRTI case definition.

|  | **Unadjusted odds** | | | | **Adjusted odds, model 2a** | | | **Adjusted odds, model 2b** | | |
| --- | --- | --- | --- | --- | --- | --- | --- | --- | --- | --- |
| **Characteristic** | **N** | **OR^a^** | **95% CI^a^** | **p-value** | **OR^a^** | **95% CI^a^** | **p-value** | **OR^a^** | **95% CI^a^** | **p-value** |
| Aged >=65y | 7,187 | 0.48 | 0.44, 0.53 | <0.001 | 0.53 | 0.48, 0.59 | <0.001 | 0.48 | 0.44, 0.54 | <0.001 |
| Male sex | 7,187 | 1.07 | 0.97, 1.17 | 0.2 | - | - | - | - | - | - |
| Dementia | 7,187 | 0.45 | 0.38, 0.52 | <0.001 | - | - | - |  |  |  |
| CCI score >4^b^ | 7,187 | 0.72 | 0.59, 0.87 | <0.001 | - | - | - |  |  |  |
| ^a^ OR = Odds Ratio, CI = Confidence Interval | | | | | | | | | | |
| ^b^ Modified Charlson Comorbidity Index, minus points for age and dementia. | | | | | | | | | | |
| Model 1b excludes dementia/ cognitive impairment and CCI score. Adjusted odds are not reported for covariates as we have not accounted for confounding of these effect estimates so results may be misleading.(2) | | | | | | | | | | |

**SUPPLEMENTARY DATA 10: Relative proportions of male and female cases by age at admission.**

**Figure S10**: Density plots showing the relative proportions of cases in the study population by age and sex. For SARS-CoV-2 positive LRTI (bottom panel) there is a peak among young females, possibly representing pregnant women who may have had a lower threshold for admission (i.e milder cases). This may have attenuated the effect of age on meeting the LRTI case definition.


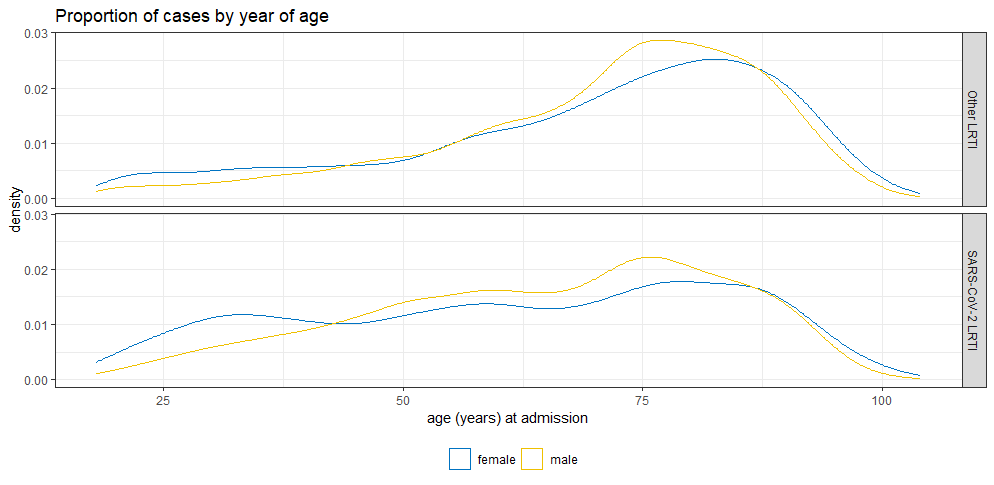


**SUPPLEMENTARY DATA 11: Log odds ratios of symptom expression comparing older with younger adults, by SARS-CoV-2 infection status.**

**Figure S11**: Comparison of LRTI symptom expression in older (≥65y) vs younger (<65y) adults is shown as a probability of each symptom occurring, with weighted log-odds ratios (empirical Bayes) expressed as z-scores.


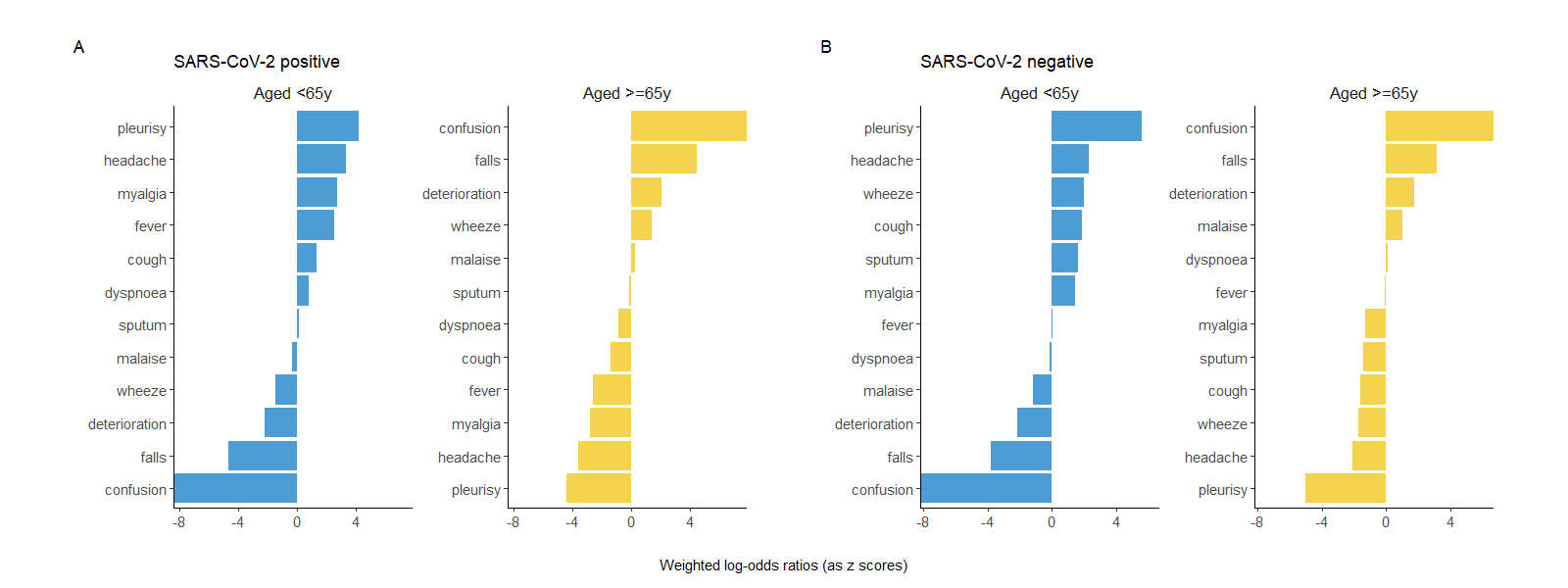


1. *National Health Service. Chest infection - NHS*. <https://www.nhs.uk/conditions/chest-infection/> (2020). [↑](#footnote-ref-2)
2. *British Medical Journal. Community-acquired pneumonia (non COVID-19) - Symptoms, diagnosis and treatment - Summary | BMJ Best Practice*. <https://bestpractice.bmj.com/topics/en-gb/3000108> (2023). [↑](#footnote-ref-3)
3. *Coronavirus disease 2019 (COVID-19) - History and exam | BMJ Best Practice.* <https://bestpractice.bmj.com/topics/en-gb/3000201/history-exam#keyFactors> (Accessed: 13 June 2023).

   ^d^ *Suspected acute respiratory infection in over 16s: assessment at first presentation and initial management*

   *NICE guideline [NG237].* <https://www.nice.org.uk/guidance/ng237/chapter/Recommendations> (2023). [↑](#footnote-ref-4)
4. Royal College of Physicians. National Early Warning Score (NEWS) 2 [Internet]. 2017 [cited 2024 Mar 15]. Available from: https://www.rcplondon.ac.uk/projects/outputs/national-early-warning-score-news-2 [↑](#footnote-ref-5)
