# Supplementary figures and images for: Syndromic case definitions for lower respiratory tract infection (LRTI) are less sensitive in older age: an analysis of symptoms among hospitalised adults
Source: BMC Infect Dis. 2024 Jun 7;24:568. doi: 10.1186/s12879-024-09425-7 (PMC11157799; doi:10.1186/s12879-024-09425-7)

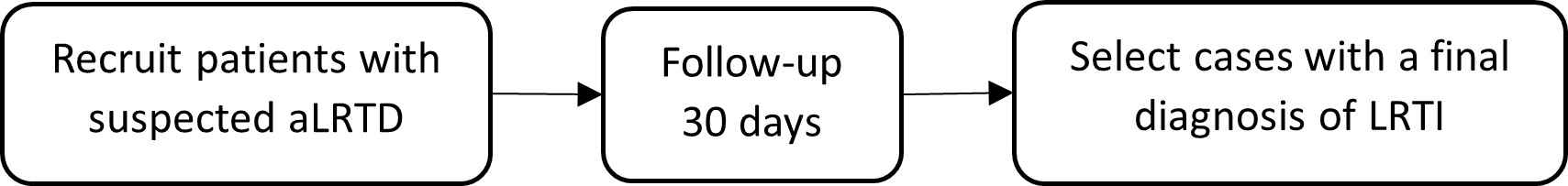

Supplement: Supplementary file 2 — Supplementary Material 2. [file 12879_2024_9425_MOESM2_ESM.jpg]

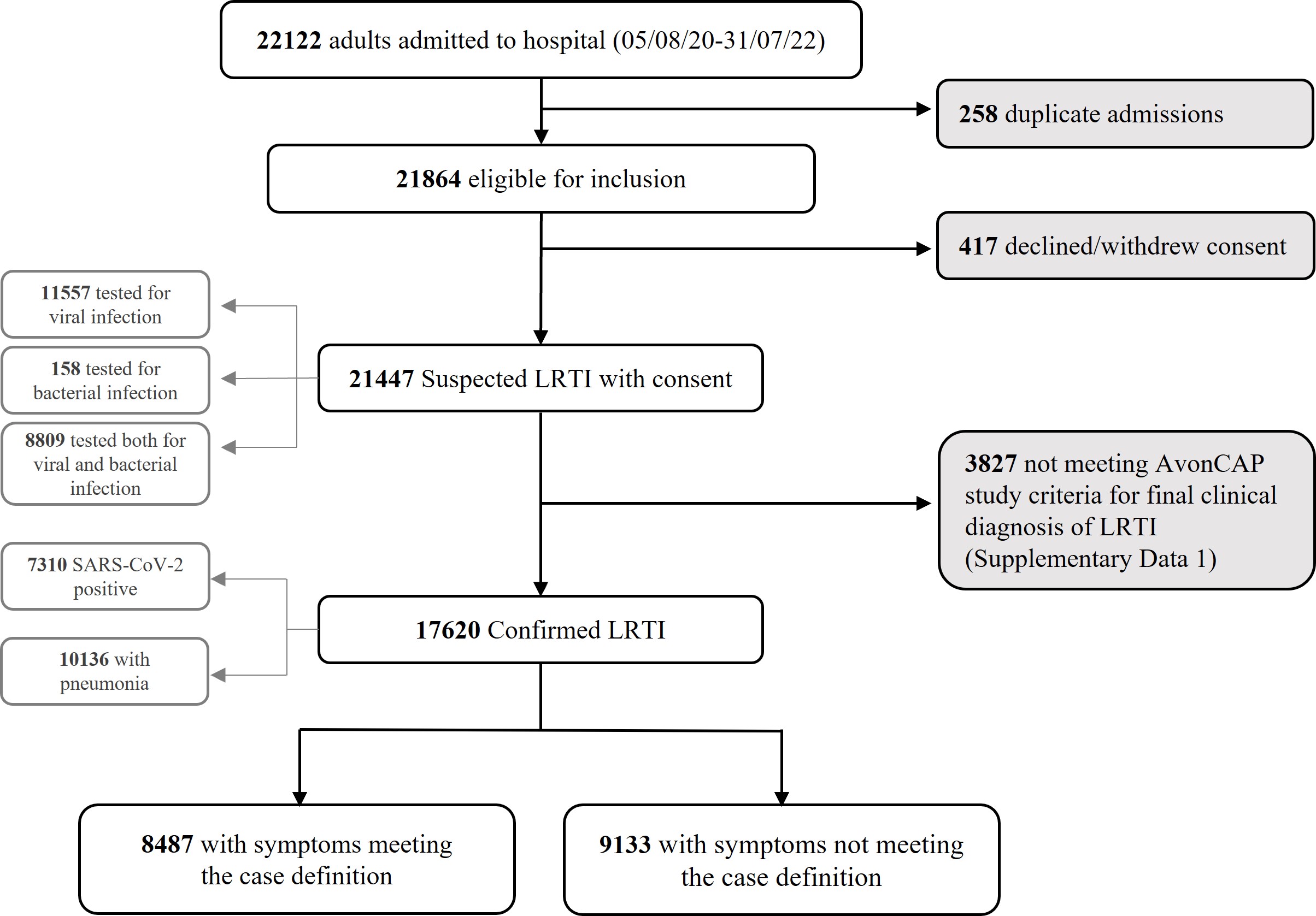

Supplement: Supplementary file 3 — Supplementary Material 3. [file 12879_2024_9425_MOESM3_ESM.jpg]

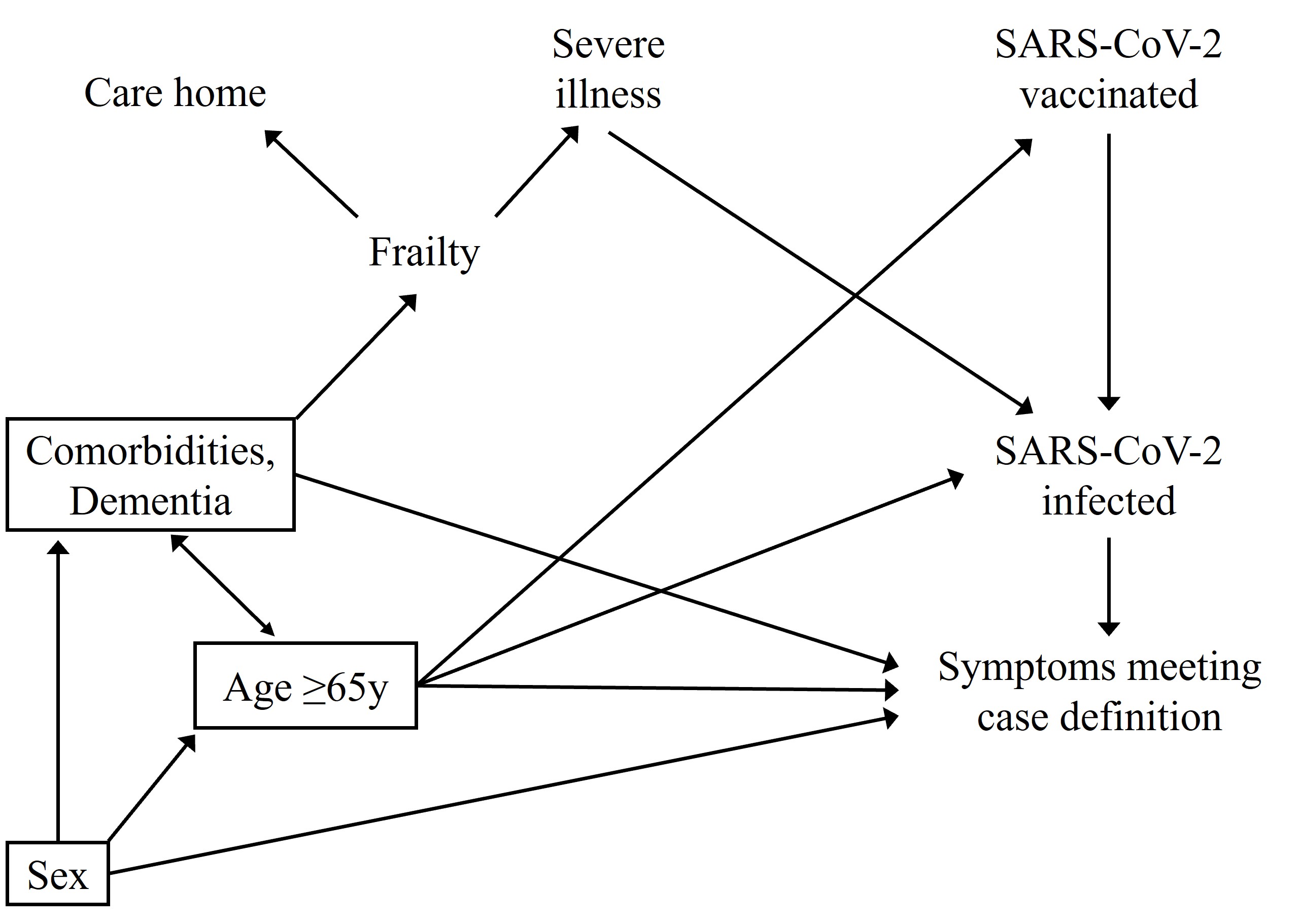

Supplement: Supplementary file 4 — Supplementary Material 4. [file 12879_2024_9425_MOESM4_ESM.jpg]

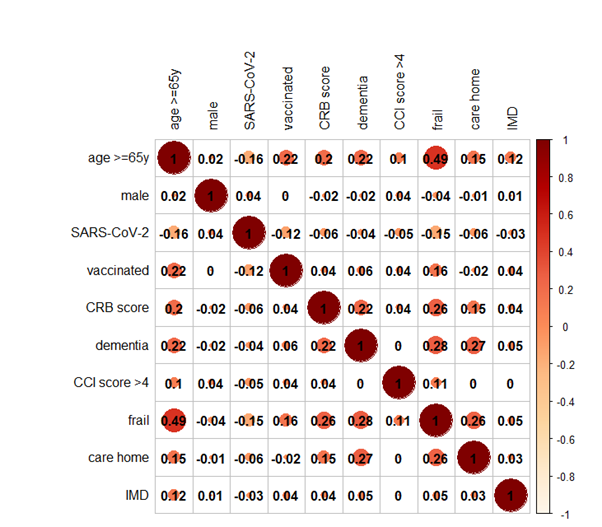

Supplement: Supplementary file 5 — Supplementary Material 5. [file 12879_2024_9425_MOESM5_ESM.tif]

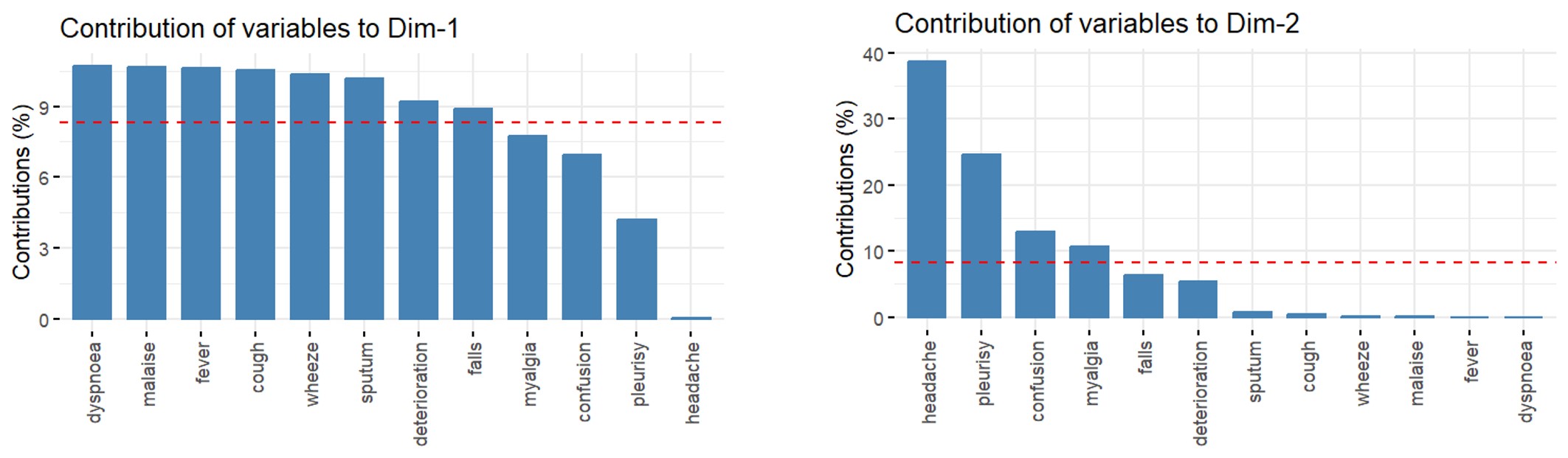

Supplement: Supplementary file 6 — Supplementary Material 6. [file 12879_2024_9425_MOESM6_ESM.jpg]

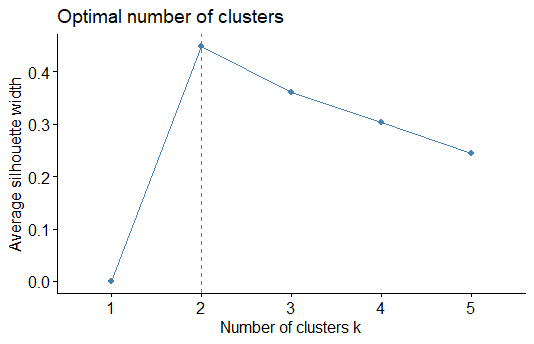

Supplement: Supplementary file 7 — Supplementary Material 7. [file 12879_2024_9425_MOESM7_ESM.tiff]
