# Supplementary material for: Syndromic case definitions for lower respiratory tract infection (LRTI) are less sensitive in older age: an analysis of symptoms among hospitalised adults
Source: BMC Infect Dis. 2024 Jun 7;24:568. doi: 10.1186/s12879-024-09425-7 (PMC11157799; doi:10.1186/s12879-024-09425-7)
Supplement: Supplementary file 10 — Supplementary Material 10. [file 12879_2024_9425_MOESM10_ESM.docx]

In this cohort, Lower Respiratory Tract Infection (LRTI) presentations varied with age and cases aged ≥65 years were significantly less likely to show symptoms indicative of LRTI. Diagnosing LRTI in older, frailer patients is challenging without laboratory or radiological investigations.
